# A case of adaptation through a mutation in a tandem duplication during experimental evolution in *Escherichia coli*

**DOI:** 10.1186/1471-2164-14-441

**Published:** 2013-07-03

**Authors:** Ram P Maharjan, Joël Gaffé, Jessica Plucain, Martin Schliep, Lei Wang, Lu Feng, Olivier Tenaillon, Thomas Ferenci, Dominique Schneider

**Affiliations:** 1 School of Molecular Bioscience, University of Sydney, Sydney, NSW, 2006, Australia; 2 Laboratoire Adaptation et Pathogénie des Micro-organismes, Université Joseph Fourier Grenoble, BP 170, Grenoble cedex 9, F-38042, France; 3 CNRS UMR5163, Grenoble cedex 9, F-38042, France; 4 TEDA School of Biological Sciences and Biotechnology, Nankai University, 23 Hongda Street, Tianjin, 300457, P.R. China; 5 Key Laboratory of Molecular Microbiology and Technology, Ministry of Education, 23 Hongda Street, Tianjin, 300457, P. R. China; 6 INSERM, UMR-S 722, Paris, F-75018, France; 7 Université Paris Diderot, Sorbonne Paris Cité, UMR-S 722 INSERM, Paris, F-75018, France

**Keywords:** Experimental evolution, Chemostats, *Escherichia coli*, Duplication, Heterozygous duplication, Insertion sequence elements, Frequency-dependent selection, Indirect selection

## Abstract

**Background:**

DNA duplications constitute important precursors for genome variation. Here we analyzed an unequal duplication harboring a beneficial mutation that may provide alternative evolutionary outcomes.

**Results:**

We characterized this evolutionary event during experimental evolution for only 100 generations of an *Escherichia coli* strain under glucose limitation within chemostats. By combining Insertion Sequence based Restriction Length Polymorphism experiments, pulsed field gel electrophoresis and two independent genome re-sequencing experiments, we identified an evolved lineage carrying a 180 kb duplication of the 46’ region of the *E. coli* chromosome. This evolved duplication revealed a heterozygous state, with one copy harboring a 2668 bp deletion that included part of the *ogrK* gene and both the *yegR* and *yegS* genes. By genetically manipulating ancestral and evolved strains, we showed that the single *yegS* inactivation was sufficient to confer a frequency dependent fitness increase under the chemostat selective conditions in both the ancestor and evolved genetic contexts, implying that the duplication itself was not a direct fitness contributor. Nonetheless, the heterozygous duplicated state was relatively stable in the conditions prevailing during evolution in chemostats, in striking contrast to non selective conditions in which the duplication resolved at high frequency into either its ancestral or deleted copy.

**Conclusions:**

Our results suggest that the duplication state may constitute a second order selection process providing higher evolutionary potential. Moreover, its heterozygous nature may provide differential evolutionary opportunities in alternating environments. Our results also highlighted how careful analyses of whole genome data are needed to identify such complex rearrangements.

## Background

Chromosomal duplications influence adaptive processes by potentially impacting on either short-term or long-term evolutionary outcomes. In the short term, duplications may confer higher fitness owing to increased gene dosage [1-3]. Indeed, they overcome limited expression of specific functions when microbial populations evolve for instance under conditions of lactose limitation in *Escherichia coli*[1,4], glucose limitation in yeast [5,6], or antibiotic pressure in *Salmonella*[2,3]. Moreover, the flexibility associated with the potential transient nature of duplication events may provide evolutionary advantages in fluctuating environments or relaxed selection [1]. Over longer evolution, duplication events can enhance survival abilities by buffering against deleterious mutations [7]. They may also provide important pathways to genetic innovation, opening opportunities for the emergence of novel functions and the expansion of gene families [6,8,9]. Genome sequences have confirmed the contribution of DNA duplications to evolutionary processes [8,10]. Gene duplication may be related to evolutionary divergence [11] by providing intermediates during the duplication-divergence process [12]. Different models have been proposed to identify the selective forces for gene duplication, including a neutral event followed by accumulation of mutations [8], the evolution of intermediates with secondary functions [13], and complementary loss-of-functions in each of the duplicated copies [14]. Chromosomal duplication is associated with the potential for increased genetic variation that may result in an equilibrium between organismal robustness against deleterious mutations and evolvability with potentially beneficial mutations that may arise in a copy of a duplicated region, thereby contributing to evolved phenotypes.

Most information on evolution through duplication relies on retrospective comparative analyses of available genomes [8]. However, direct observation of the rise and segregation of duplications is much scarcer owing to their rarity, instability and the subtle phenotypic effects they may confer. Bacterial evolution experiments [15] provide opportunities to identify intermediates during duplication processes. However, only few duplication events have been detected in these experiments, except during high temperature adaptation [16] where evolved clones contained on average 0.2 large duplication compared to 9.2 SNPs and short indels [17]. Most mutations identified during laboratory evolution experiments include SNPs and short indels [18-21] that have been shown to explain many evolved phenotypes. Consequently, little information is available over short time-frames on the dynamics of duplication processes, including the divergence of duplicated copies. Hence, to the best of our knowledge, no data with co-linear wild-type and mutant alleles are available during evolution experiments, whereas duplications have been commonly found by classical genetics in *Salmonella* populations adapting to diverse conditions including nutrient-limited chemostats [3,22].

We designed evolution experiments during which an *E. coli* ancestral strain was propagated as large populations (>10^10^ bacteria) for ~100 generations within chemostats under glucose or phosphate limitation [23,24]. Chemostats are continuous culture systems in which the bacteria deplete substrates but are maintained in constant population numbers over extended periods. Mutations improve fitness in this environment, allowing population sweeps. The initial selection is for better utilization (transport and scavenging) of limiting nutrient but, after ~50 generations, populations diversify into separate lineages with diverse phenotypes and eventually evolve cross-feeding polymorphisms [19,23-26]. Many SNPs, deletions, IS movements and short indels have been identified that explained the functional changes that occurred in different phenotypic groups [19,27]. However, the genetic bases of the metabolic changes and fitness benefits of some groups of clones have not yet been identified. In particular, one group of clones producing unusually elevated acetate levels and reduced growth yield is not characterized by the typical improved glucose transport [28] and lacks all the regulatory changes identified so far in other clones from the same populations [23].

Here, we combined Insertion Sequence-based Restriction Length Polymorphism, pulsed-field gel electrophoresis and genome re-sequencing to characterize one representative of this group of clones. We identified a novel chromosomal duplication, one copy of which was altered by a deletion that conferred higher fitness under the conditions prevailing in the chemostat. This duplication may constitute an evolutionary intermediate [12]. Hence, the duplication is readily resolved into a single copy, either ancestral or evolved, the resolution frequency being higher under non-selective conditions. Owing to its instability, we had difficulties to detect the duplication by genome re-sequencing. Hence, we missed the duplication during a first genome re-sequencing attempt of the focal clone. Only the discrepancy between the data generated by our combined approaches led us to carefully re-examine the genome and to identify the duplication. This instability may explain the under-representation of large duplications observed during laboratory bacterial evolution experiments.

## Results

### Initial characterization of BW4005

**Figure 1:**
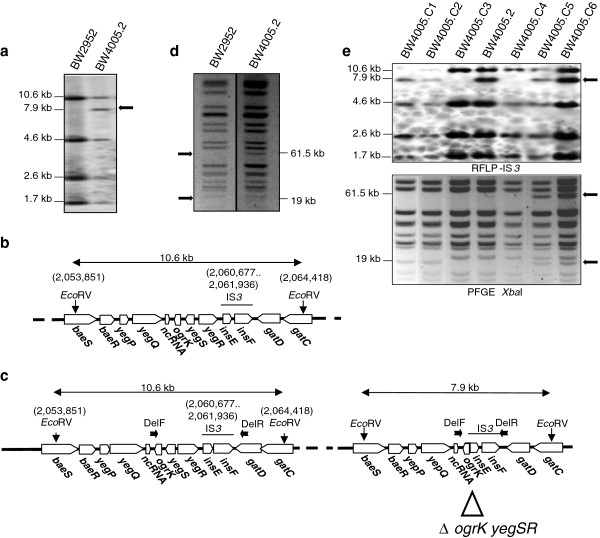
**Genomic rearrangements in the evolved clone BW4005 sampled from a glucose-limited chemostat. (a)** RFLP-IS*3* of *Eco*RV-digested genomic DNA of BW2952 and BW4005.2 [24]. A fifth hybridizing fragment (arrow) with stronger hybridization intensity was detected in BW4005.2. **(b,c)** Maps showing the genomic regions corresponding to the 10.6 and 7.9 kb *Eco*RV IS*3*-hybridizing fragments, including the sequences adjacent to IS*3* in BW2952 **(b)** and BW4005.2 **(c)**. Genomic coordinates (given in parentheses) and gene orientations (horizontal boxes) are based on the genome sequence of BW2952 [29]. Vertical arrows indicate *Eco*RV restriction sites, and horizontal arrows show locations of primers used to identify the junction of the duplicated region (Table 2). The triangle shows the location of the 2668-bp deletion in BW4005.2. **(d)** PFGE of *Xba*I-digested genomic DNA of BW2952 and BW4005.2. The additional bands in BW4005.2 compared to BW2952 are shown by arrows. **(e)** RFLP-IS*3* and PFGE profiles of six clones (BW4005.C1 to BW4005.C6) derived from the initial glycerol stock of BW4005. The sizes of relevant bands are indicated on the left.

The evolved clone BW4005 was previously characterized phenotypically by comparison to its ancestor, and exhibited a lower growth yield, a higher production of acetate from glucose in chemostat culture, and a lack of obvious difference in glucose uptake [28]. At the ecological level, BW4005 was present in low proportion in its glucose-limited evolving population and displayed negative frequency-dependent fitness when competed with the ancestor [19]. By contrast, no genetic information was available to explain these phenotypic and ecological traits, even after re-sequencing the genome of a clone, called BW4005.1, recovered from the initial glycerol stock of BW4005, by using a combination of 454 and Illumina technologies. Indeed, we detected a single mutation compared to the ancestor, consisting of a short deletion in *ahpC* encoding the alkyl hydroperoxide subunit involved in peroxide resistance (Table 1). As shown below, this mutation did not contribute to increased fitness in the chemostats. Moreover, the genome sequence of BW4005.1 was inconsistent with previously detected changes in the RFLP profiles of another clone isolated independently from the same glycerol stock of BW4005, called BW4005.2, using the mobile genetic element IS*3* as a probe [27]. Indeed, while the RFLP-IS*3* profile of the ancestral strain BW2952 revealed four IS*3*-containing *Eco*RV genomic fragments of 1.7, 2.6, 4.6, and 10.6 kb, respectively, with hybridization intensities proportional to the relative molecular weight of these fragments (Figure 1a), the one of BW4005.2 showed a fifth hybridizing fragment of ~7.9 kb with a hybridization intensity stronger than expected from its molecular weight (Figure 1a). This was unlikely to be due to contamination, since both clones harbored the *ahpC* mutation (Table 1).

**Table 1:** List of mutations found in BW4005.1 and BW4005.C6

**Event number**	**Genome position**^**a**^	**Gene name**	**Type**	**Present in**^**b**^
1	541,030	*ahpC*	3-bp deletion	BW4005.1
				BW4005.C6
2	2,058,010-2,060,677	*ogrK-yegS*-*yegR*	2668-bp deletion	BW4005.C6
3	1,993,429-2,172,740	NA^c^	~180-kb duplication	BW4005.C6

^a^The positions are given according to the genome sequence of the ancestor strain BW2952 [29]. ^b^The genome sequence of BW4005.1 was first obtained and detected only the first mutation, while the one of BW4005.C6 was obtained afterwards and contained the three mutations. ^c^Not applicable.

### Genomic rearrangements in BW4005

We characterized the genomic sequences adjacent to IS*3* in the 7.9-kb fragment specific to BW4005.2 by inverse PCR experiments. One side (right on Figure 1b) corresponded to the *gatD* gene with the IS*3* insertion at exactly the same position (2,061,936) as observed in the ancestor for the IS*3* element contained in the 10.6-kb hybridizing fragment. This particular ancestral IS*3* is inserted between positions 2,060,677 and 2,061,936. The other side corresponded to a genomic sequence (2,058,010) located 2668 bp upstream in the ancestral genome. Therefore, this fifth IS*3* probably resulted from an IS*3* transposition event at position 2,058,010 followed by a recombination event between this new IS*3* copy and the one located in the ancestral genome between positions 2,060,677 and 2,061,936 within *gatD*. These events subsequently resulted in the 2668-bp deletion of the intervening fragment, affecting the two genes *yegR* and *yegS* and part of *ogrK* (Figure 1c). However, two features of the RFLP-IS*3* pattern suggested a more complex rearrangement (Figure 1a): the two 10.6 and 7.9 kb IS*3*-hybridizing fragments were co-existing in BW4005.2, indicating the presence of two copies of at least *gatD*, and the 7.9 kb fragment presented a hybridizing intensity higher than expected from its molecular weight compared to the four other hybridizing fragments. Altogether, these data might be explained by the presence in the chromosome of BW4005.2 of a tandem duplication containing two heterozygous copies, with and without the IS*3*-generated deletion respectively (Figure 1c). We then performed PFGE experiments with *Xba*I-digested genomic DNA of BW2952 and BW4005.2 (Figure 1d). We found two extra *Xba*I fragments of 61.5 and 19 kb in BW4005.2 compared to BW2952, which also suggested the presence of a complex rearrangement.

These genomic rearrangements were not detected by genome sequencing in the clone BW4005.1. Since both clones BW4005.1 and BW4005.2 were derived from a single cycle of growth after plating the initial glycerol stock of BW4005 onto LB solid medium, we hypothesized that the rearrangements were present in the original evolved clone, but were characterized by a high instability. We therefore investigated the stability of the genomic rearrangements by isolating six additional clones, called BW4005.C1 to BW4005.C6, after plating the initial glycerol stock of BW4005 onto LB medium, and analyzing them by RFLP-IS*3* and PFGE (Figure 1e). The RFLP-IS*3* profiles were checked for the presence or absence of the 10.6 and 7.9 kb IS*3*-hybridizing fragments. Hence, their simultaneous presence was diagnostic of the putative heterozygous duplication while the presence of only the first or second fragment indicated the resolution of the putative duplication into the ancestral or deleted copy, respectively. Similarly, the PFGE profiles were checked for the presence or absence of the two 61.5 and 19 kb bands. The RFLP-IS*3* profiles revealed that four (BW4005.C1 to BW4005.C4) of the six clones had lost either one of the putative duplicated DNA copies (Figure 1e), with clones BW4005.C1 and BW4005.C2 harboring the single deleted copy and BW4005.C3 and BW4005.C4 the ancestral copy. The two last clones, BW4005.C5 and BW4005.C6, retained the putative heterozygous duplication. Consistent with the RFLP-IS*3* profiles, the PFGE analysis showed that clones BW4005.C1 and BW4005.C2 retained only the 19 kb band, while BW4005.C3 and BW4005.C4 had the ancestral profile, and BW4005.C5 and BW4005.C6 harbored the two extra fragments (Figure 1e). These results indicated the high genetic instability of the putative duplication as well as its capacity for resolution into alternative states.

Moreover, both RFLP and PFGE experiments, using genomic DNA isolated from different independent cultures of BW4005.C6, exhibited variation in the intensity of the additional bands (Figure 1 and data not shown), which further suggested the instability of the genetic rearrangement. This instability may provide an explanation for the discrepancies between the RFLP/PFGE experiments and the genome re-sequencing results and is considered in more detail below.

### Characterization of a heterozygous duplication by genome re-sequencing

**Figure 2:**
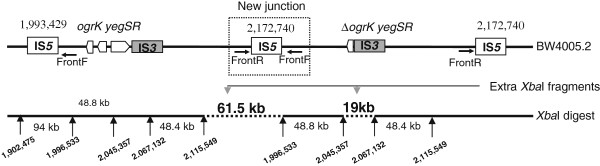
**Map of the genomic rearrangement in the 46’ region of the BW4005 chromosome.** The top map shows both the ~180 kb duplication of the ancestral chromosomal region, that extends between the two IS*5* elements located at positions 1,993,429 and 2,172,740, and the relevant IS*3* elements contained within that duplication. The copy on the right carries the 2668-bp deletion containing part of *ogrK* and all of *yegR* and *yegS*. The bottom line shows the restriction map derived from the PFGE profile of *Xba*I-digested genomic DNA of BW4005.2. Vertical arrows indicate locations of *Xba*I sites and horizontal lines with arrows indicate the locations of primers used for the identification of the new junction generated by the duplication (shown in the box). The sizes of the different *Xba*I fragments are indicated between the vertical arrows. For gel documentation of the BW4005.2 PFGE profiles showing the extra *Xba*I bands of 61.5 and 19 kb (indicated by dashed lines), see Figure 1d,e. Genome positions of *Xba*I restriction sites are based on the genome sequence of BW2952 [29].

To address the discrepancies between the different approaches, we decided to re-sequence the genome of BW4005.C6 that harbored the complex rearrangement (Figure 1e). We now detected three mutations compared to BW2952 (Table 1): the same in-frame 3-bp deletion in *ahpC*, the 2668-bp deletion of *ogrK*-*yegR*-*yegS*, and a duplication of ~180 kb extending from genomic positions 1,993,429 to 2,172,740. The duplicated fragment is localized between two IS*5* elements (Figure 2), and therefore likely resulted from a recombination event between one and the other of these two IS*5* copies during DNA replication. We further validated the presence of the duplication by three additional tests: first, we PCR-amplified and sequenced the new junction sequence resulting from the duplication by using the primers FrontR and FrontF (Table 2, Figure 2). Both the size and sequence of the PCR products confirmed the duplication. Second, an additional IS*5*-containing *Eco*RV restriction fragment of 1.5 kb should be produced at the junction of the duplication (Figure 2) and should therefore be detected by RFLP-IS*5*. This was indeed shown to be the case (data not shown). Finally, the PFGE profiles with the additional *Xba*I bands were easily explained on the basis of the duplicated genome sequence (Figure 2).

**Table 2:** List of primers used in this study

**Primer name**	**Direction**	**Genome position**^**a**^	**Sequence (5'-3')**^**b**^	**Used for**
IS*3*F1	Forward	2,057,888	CGCTGTACCGACTCATAAGT	Detection of *ogrK*-*yegSR* deletion
IS*3*R1	Reverse	2,061,063	GATGCTGAACTCAGCCTGATG	Detection of *ogrK*-*yegSR* deletion
IS*3*R2	Reverse	2,059,299	CATTCCTTCCTCACGCAAC	Detection of *ogrK*-*yegSR* deletion
IS*5*F1	Forward	2,172,688	CACCATCAACTGTCTCACCA	Detection of duplication
IS*5*R1	Reverse	1,993,645	GACCCGCAGATGATGATTAC	Detection of duplication
DelF	Forward	2,057,748	CACCGTAACGCTGTTTTGACCG	Detection of *ogrK*-*yegSR* deletion
DelR	Reverse	2,062,009	GGATCTTGAGCTCAATTACGCGC	Detection of *ogrK*-*yegSR* deletion
IS*3*EF	Forward	NA^c^	GCTGCTACGATAATGCCTGCG	Inverse PCR
IS*3*ER	Reverse	NA^c^	GCGTTCAGCAAGCTTCAGGG	Inverse PCR
FrontR	Forward	1,993,555	GTACATTATGCCTGTTCCGAG	Detection of duplication
FrontF	Reverse	2,172,636	TCGTATTATTGGCGGTCCC	Detection of duplication
*ogrK*AmpF1	Forward	2,057,979	GGCTTGTACGCGCATGTGCGGCATGCTGGCATA**AGAGTTGGTAGCTCTTGATC**	Strain construction
*yegS*AmpF2	Forward	2,058,495	CTCACTCTCCACATTTGAATGTCAGACGAGCGAC**AGAGTTGGTAGCTCTTGATC**	Strain construction
*yegR*AmpF3	Forward	2,059,218	CATGGCAGAATTTCCCGCCAGCTTACTGATTCTT**AGAGTTGGTAGCTCTTGATC**	Strain construction
*ogrK*AmpR1	Reverse	2,058,472	GGTAAGCGTCAAATATGCGCGTTCTGGCTGTGC**CATTCAAATATGTATCCGCT**	Strain construction
*yegS*AmpR2	Reverse	2,059,189	TGATTGGTCTGTAGATAGTGTAGAGCAGAAAAC**CATTCAAATATGTATCCGCT**	Strain construction
*yegR*AmpR3	Reverse	2,060,666	GACCGTTATTCTCGACAGCGGAAGTACGACAATG**CATTCAAATATGTATCCGCT**	Strain construction

^a^The positions are given according to the genome sequence of the ancestor strain BW2952 [29]. ^b^Sequences in bold are homologous to the *bla* antibiotic resistance gene. ^c^Not applicable; these primers are complementary to sequences inside the IS*3* elements.

### Phenotypic effects of the rearrangements

We next investigated the relationships between the complex chromosomal rearrangements and the phenotypes known for BW4005. We measured the growth yield, acetate production, and fitness of the three clones BW4005.C2, BW4005.C3 and BW4005.C6 compared to the ancestor BW2952. These three clones are genotypically different (Figure 1e): the duplication has been resolved into the deleted or ancestral copy in BW4005.C2 and BW4005.C3, respectively, while BW4005.C6 contains the heterozygous duplication. As shown in Table 3, BW4005.C2 and BW4005.C6 had similar evolved phenotypes (decreased fitness at a 50:50 ratio, reduced yield, and high acetate production under glucose limitation), while BW4005.C3 had phenotypic properties similar to the ancestor. These results suggested that the 2668-bp deletion was the main contributor to the BW4005 evolved phenotypes, and not the *ahpC* mutation. Moreover, the presence of the heterozygous duplication seemed to confer similar phenotypes compared to the 2668-bp deletion alone.

**Table 3:** **Phenotypic traits of ancestral, evolved and reconstructed *E. coli *strains**

**Strains**	**Description**	**Yield**^**a**^	**Acetate production**^**b**^	**Fitness (*S *h**^**-1**^**)**^**c**^	
				**50:50**	**1:99**
BW2952	Ancestor	1.00	-	1.00	1.00
BW4005.2	Derivative of evolved isolate BW4005	0.89 ± 0.02	+	−0.04 ± 0.01	0.06 ± 0.01
BW4005.C2	Derivative of BW4005 (DO)^d^	0.74 ± 0.08	+	−0.09 ± 0.01	ND
BW4005.C3	Derivative of BW4005 (WT)^d^	0.98 ± 0.03	-	−0.02 ± 0.01	ND
BW4005.C6	Derivative of BW4005 (HD)^d^	0.83 ± 0.05	+	−0.04 ± 0.01	ND
BW6029	BW2952 *yegS*::*bla*	0.85± 0.03	+	−0.02 ± 0.01	0.09± 0.04
BW6030	BW2952 *yegR*::*bla*	1.01 ± 0.02	-	0.01 ± 0.01	−0.01 ± 0.01
BW6031	BW2952 ∆*ogrK-yegS*-*yegR*::*bla*	0.88 ± 0.02	+	−0.05 ± 0.01	0.11± 0.04

^a^Growth yields are shown relative to the ancestral strain BW2952 and were obtained from 24-hour glucose-limited chemostats by measuring the optical density of the cultures at 600 nm. Values are from three independent measurements.

^b^Acetate production was tested with 24-hour chemostat cultures. +, acetate present at 2–5 mg l^-1^; -, below the detection limit (< 0.15 mg l^-1^). Data were from at least three independent biological repeats with less than 10% standard errors.

^c^Competing strains were mixed at 50:50 or 1:99 ratios after individual acclimation in the chemostat evolution conditions. Changes in the proportions of the competitors after 24 hours were used to calculate fitness values. Data presented are mean ± SEM from at least two independent experiments. ND, not determined.

^d^Symbols are identical to the legend of Figure 3.

We next investigated the respective phenotypic contribution of each of the *yegR* and *yegS* genes, by first moving the 2668-bp deletion and second inactivating them individually into the ancestral genetic background. We found that both the 2668-bp deletion and *yegS*-inactivated strains exhibited the evolved phenotypes, while the *yegR*-inactivated strain had phenotypes indistinguishable from the ancestor (Table 3).

### Stability of the duplication

**Figure 3:**
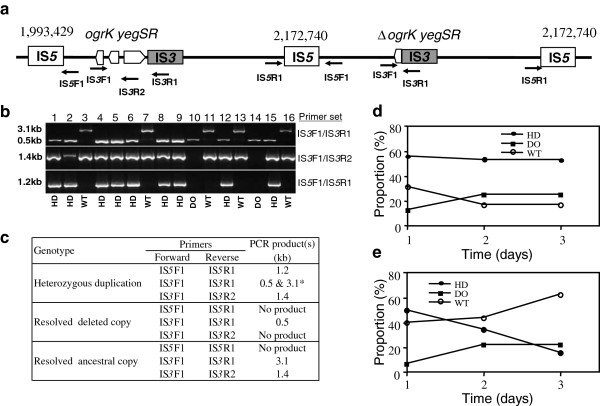
**Stability of the duplication and phenotypes of the different derived genotypes. (a)** Chromosomal map of the duplication in BW4005 showing primer pairs used to follow the fate of the duplication. The same symbols as in Figure 2 are used. **(b)** Example of PCR experiments to score the state of the genomic rearrangement, by using the three primer pairs IS*5*F1/IS*5*R1, IS*3*F1/IS*3*R1 and IS*3*F1/IS*3*R2. WT and DO stand for the presence of the single ancestral or 2668-bp deleted copy, respectively, and HD for the presence of the heterozygous duplication. The expected results for each of the three genotypes are shown in **(c)**. * Owing to the higher efficiency of production of the smaller 0.5 kb PCR product with IS*3*F1/IS*3*R1, we had difficulties in detecting simultaneously the larger 3.1 kb PCR product in clones harboring the heterozygous duplication. We therefore also performed PCR reactions using the additional primer pair IS*3*F1/IS*3*R2 to improve the accuracy of scoring the heterozygous duplication state. **(d,e)** Stability of the heterozygous duplication under the selective conditions of the chemostat **(d)** and non-selective conditions of LB batch cultures **(e)**. In **(d)**, BW4005.C6 harboring the heterozygous duplication was mixed 1:99 with the MC4100 reference strain after individual acclimation in separate chemostats. Samples were taken every 24 hours for three days and appropriate dilutions were plated onto LB plates with or without 30 μg ml^-1^ kanamycin. At each time point, we sampled 32 well-separated colonies from Kan-plates and scored them for the IS*5*-mediated duplication and IS*3*-mediated deletion. In **(e)**, an overnight culture of BW4005.C6 was diluted 100-fold into LB liquid medium and grown for 24 hours. This growth cycle was repeated for two additional days and the proportion of the different genotypes was followed as in **(b)**. The data presented are the average of two independent cultures in each case.

An important unresolved question is whether the duplication itself conferred a benefit under the chemostat selection conditions. Since evolved phenotypic attributes were not specifically provided by the duplicated state, we tested this indirectly by comparing the population dynamics of the duplication in two environments: non-selective conditions in LB batch cultures and the glucose-limited chemostat selection environment (Figures 1e and 3). The clone BW4005.C6, harboring the heterozygous duplication, was grown either in LB medium for three 24-hour growth cycles or for three days in chemostats in low-frequency competition with MC4100 to mimic the evolution experiment (Materials and methods). In each case, 32 individual clones, sampled at each day interval, were analyzed for the presence of the genomic rearrangements by colony-PCR using the primer pairs IS*5*F1/IS*5*R1, IS*3*F1/IS*3*R1, and IS*3*F1/IS*3*R2. The primer pair IS*5*F1/IS*5*R1was specifically designed to detect the new junction produced by the duplication, and IS*3*F1/IS*3*R1 and IS*3*F1/IS*3*R2 to detect the 2668-bp IS*3*-mediated deletion (Figure 3a-c). Under the non-selective conditions (Figure 3e), the proportion of clones harboring the heterozygous duplication decreased significantly by the third 24-hour growth cycle (*t*-test: *P* < 0.05), with the concomitant significant increase of the proportion of clones with single ancestral or deleted copies (*t*-test: *P* < 0.05). By contrast, the proportion of clones harboring the heterozygous duplication was high and stable over the three days under the original selection condition (Figure 3d). These results showed the genetic instability of the heterozygous duplication under non-selective conditions, in stark contrast to its stability over a short-term period in the glucose-limited conditions prevailing during the evolution experiment. However and despite these stability differences, we were unable to distinguish phenotypically (Table 3) the clones harboring the heterozygous duplication and the single deleted copy (even the reconstructed clone with the specific *yegS* inactivation in the ancestral genetic background had identical evolved phenotypes).

## Discussion

Evolution experiments propagating bacterial populations in glucose- or phosphate-limited chemostats have been extensively developed to understand the dynamics of adaptive processes [19,24-26,28]. Genes involved in transport and consumption of glucose [30-32], encoding global regulators of gene expression [33,34] and DNA repair enzymes [27,35] have been shown to be changed during evolution. However, several evolved clones followed alternative evolutionary pathways not uncovered by whole-genome sequencing. Here, we identified one such path, consisting of complex chromosomal rearrangements that included a large 180-kb duplication of the 46’ chromosomal region followed by the divergence of the two tandem duplicated copies. One copy experienced a 2668-bp deletion removing part of the *ogrK* gene and both *yegR* and *yegS*. We showed that the duplication was highly unstable under non-selective conditions but not in the conditions prevailing during evolution. However, the duplication per se did not provide any of the phenotypic traits associated with the adaptation of these evolved clones in the chemostats. In striking contrast, inactivating *yegS* in the ancestral background was sufficient to confer all evolved phenotypes.

The benefit conferred by the *yegS* deletion, together with the dominance of the deleted allele over its ancestral counterpart, are difficult to infer owing to the lack of information about YegS. Indeed, YegS is annotated as a putative diacylglycerol kinase potentially involved in the response to acid stress [36]. A *yegS* knockout results in the loss of mannose and acetate consumption [37]. Diacylglycerol is a lipid second messenger in many organisms [38], and the *yegS* deletion may result in alterations of regulatory and/or metabolic networks owing to changes in lipid signaling pathways in *E. coli*.

Previous studies, with *Salmonella typhimurium* populations propagated in arabinose- or melibiose-limited chemostats [22], detected 96 and 25%, respectively, of cells harboring *araE* or *melB* duplications that emerged within only 50–100 hours of incubation. The amplified genes are responsible for arabinose or melibiose uptake and are therefore under strong selection for elevated expression levels under these conditions. In *Salmonella*, duplications and rearrangements also occur upon prolonged storage of strains and in clinical settings [39,40]. In contrast, in the glucose-limited chemostat populations of *E. coli*, the major early adaptive changes involve a series of regulatory mutations increasing the levels of glucose transport components [19,24-26,28,41]. Earlier studies of lactose-limited chemostats also showed that the first selective sweeps were by *lac*-constitutive mutants, only later followed by DNA amplifications [4]. Therefore, one may speculate whether inherent differences between *Salmonella* and *E. coli* may exist for duplication rates and/or costs. Interestingly, the BW4005 lineage carrying the duplication emerged later as a minority clone, with an adaptive pathway very different from the other clones in the chemostat population [19], since its fitness was shown to be frequency dependent.

While many duplication (and amplification) events have been detected in classical genetic studies where strong selection was applied, it is not the case during laboratory evolution experiments, with one notable exception [16,17]. We showed here that this may be merely linked to the high instability of the duplication events under non-selective conditions. Hence, a single overnight growth cycle was sufficient to generate at high frequency offspring that lost the duplication (Figure 1e). Most analyses of evolved clones sampled from evolution experiments involve culture cycles under non-selective conditions for further analyses. We show here that such experimental practices may result in the loss of duplication events and that sampling single clones may lead to an underestimation of these chromosomal rearrangements. Therefore, storing and analyzing evolved isolates must be carefully performed. Instabilities in bacterial stocks are well-known [39,42,43] and our data emphasize the importance of minimizing sub-culturing steps. It was only good fortune that at least one clone, randomly sampled from the original chemostat population, retained the duplication.

A comprehensive view of the stability of genome duplications is yet missing at the evolutionary, ecological and molecular levels. The RecA protein is involved in homologous recombination and is important in resolving duplications [44]. On the other hand, some bacterial duplications may be stable, including those involving recombination at rRNA (*rrn*) operons [39,45]. Here, we observed that the heterozygous duplication was much more stable under the glucose-limited chemostat conditions than under non-selective conditions (Figure 3). Therefore, it might confer a direct selective advantage in the evolution regime. However, all evolved phenotypes were related to either the 2668-bp deletion alone or the single inactivation of *yegS*. Hence, the duplication by itself does probably not confer a direct benefit to the evolved cells, in contrast to duplications that result in a gene-dosage advantage [1-3].

Several reasons may explain the stability differences in selective and non-selective conditions. First, the resolution components may be differentially regulated in various environments. Second, the fitness cost of the duplication may be larger during rapid growth resulting in higher instability. Moreover, we showed that the heterozygous duplication had no fitness disadvantage over the single deletion copy, suggesting little fitness cost in chemostats. Third, the duplication may represent a precursor enhancing the likelihood of the substitution of a beneficial mutation through an increase in the mutational target and in local mutation rates. As such, the contribution of the duplication in the evolution of BW4005 may involve a second-order selection process [46] that has been shown to occur during experimental evolution with *E. coli*[47]. Finally, our results are not consistent with the model for divergence after duplication [2], in which selection continuously favors both the maintenance of the duplicated copies and the divergence of one copy from the parental one.

A fascinating property of the evolved duplication is its ability to resolve into either the ancestral or deleted copy that may provide robustness by buffering against deleterious mutations. On the other hand, the capacity to recover the ancestral chromosome when selection is removed endows the evolved strain with increased evolvability in alternative environments. Finally, the demonstration of ancestral and evolved copies inside a duplication provides a newly documented example of an evolutionary intermediate, with different selection pressures acting on the two copies providing the basis of evolutionary divergence.

## Conclusions

We identified for the first time during laboratory evolution experiments the presence of a heterozygous duplication, one copy presenting a small deletion that was shown to be dominant and beneficial under the selective conditions. Studies of the stability of the duplicated genotype under different environmental conditions revealed that it was unlikely to have been submitted to direct selection during evolution but rather to indirect selection. This duplication therefore balances the robustness and evolvability traits of the bacterial populations evolving in the glucose-limited chemostats.

## Methods

All bacterial strains used in this study are listed in Table 4. The *E. coli* K-12 strain BW2952 [29], a kanamycin-resistant derivative of MC4100, was used as the ancestor to initiate glucose-limited chemostats that were fed with 0.02% wt/vol glucose in minimal medium A [48] with a 0.1 h^-1^ dilution rate, as previously described [49]. This study focused on the BW4005 evolved strain, one of 41 randomly sampled clones from the 26-day sample (representing ~90 generations of evolution) of one such chemostat [24]. These 41 evolved clones were preserved as glycerol suspensions at −80°C directly after sampling. During this study, eight clones, called respectively BW4005.1, BW4005.2 and BW4005.C1 to BW4005.C6, were directly derived from the initial glycerol stock of BW4005.

**Table 4:** List of strains used in this study

**Strain**	**Relevant genotype**	**Source**
MC4100	F^-^*araD139* ∆(*argF-lac*)*U169 rspL150 deoCl relA1 thiA ptsF25 flb5301 rbsR*	[29]
BW2952	MC4100 *malG*::λ*plac*Mu55f(*malG*::*lacZ*)	[29]
BW3454	MC4100 *metC162*::Tn*10*	[25]
BW4005	Chemostat evolved clone sampled after 90 generations	[19,24]
BW4005.C1	Colony 1 derived from BW4005	This study
BW4005.C2	Colony 2 derived from BW4005	This study
BW4005.C3	Colony 3 derived from BW4005	This study
BW4005.C4	Colony 4 derived from BW4005	This study
BW4005.C5	Colony 5 derived from BW4005	This study
BW4005.C6	Colony 6 derived from BW4005	This study
DY330	W3110 ∆*lacU169 gal490 λcl857* ∆(*cro-bioA*)	[50]
BW6026	DY330 *yegS*::*bla*	This study
BW6027	DY330 *yegR*::*bla*	This study
BW6028	DY330 *ogrK-yegS-yegR*::*bla*	This study
BW6029	BW2952 *yegS*::*bla*	This study
BW6030	BW2952 *yegR*::*bla*	This study
BW6031	BW2952 ∆*ogrK-yegS-yegR*::*bla*	This study

Competitions in chemostats were performed between the kanamycin-resistant, tetracycline-sensitive evolved clone BW4005.C6 derived from BW2952 and the kanamycin-susceptible tetracycline–resistant BW3454 strain, which was used as the common reference ancestral competitor.

Outside chemostats, strains were grown in LB liquid or solid medium. Allelic exchange was performed using LB+sucrose plates without NaCl and supplemented with 50% sucrose. When necessary, antibiotics were added: chloramphenicol or kanamycin each at 30 μg ml^-1^, and ampicillin at 100 μg ml^-1^.

### Strain construction

The 2668-bp deletion from the BW4005 evolved clone was moved into the ancestral genome by allelic exchange using the suicide plasmid pKO3 [51]. Briefly, a 1100-bp PCR fragment with about 550 bp of adjacent sequences on each side of the deletion was obtained using BW4005 genomic DNA and the primers DelR and DelF (Table 2), and then cloned into pKO3. The obtained plasmid was introduced by electro-transformation into BW2952 and chloramphenicol-resistant cells, formed after chromosomal integration of the plasmid, were selected. Resistant clones were streaked onto LB+sucrose plates to select for cells that lost the plasmid. Indeed, pKO3 carries the *sacB* gene that makes cells carrying that plasmid susceptible to killing by sucrose. These cells were then screened by PCR and scored for the presence of the deletion allele by the size of the PCR products. We constructed *yegS*::*bla* and *yegR*::*bla* derivatives of BW2952 using homologous flanking sequences as described [50]. The primer sets used for strain constructions are listed in Table 2.

### Phenotypic assays

Analysis of extracellular acetate in culture media from individual chemostats inoculated with different bacterial strains was performed after filtration through a Milex® filter unit (Millpore, Ireland, 0.22 μm) by using an assay kit for acetic acid with the UV method (Boehringer Mannheim/R-Biopharm, Germany), according to the manufacturer’s guidelines. The growth yields were inferred by measuring the optical density at 600 nm of 24-hour chemostat cultures growing in the exact same conditions prevailing during the evolution experiment.

### Pairwise competition experiments and fitness estimation

Each bacterial strain was competed against a *metC*::Tn*10* derivative, called BW3454 (Table 4), of the BW2952 ancestral strain [29], under the same conditions used during the chemostat evolution experiment, except that the medium was supplemented with 4 μg ml^-1^ methionine. Before mixing the two competitors, each strain was independently acclimated by overnight growth in the glucose-limited chemostats to ensure identical physiological state. The fitness of the competing strains was calculated as the selection coefficient *S*, as described [19].

### Genome re-sequencing

A first single colony of BW4005, called BW4005.1, was isolated after streaking the original glycerol suspension on LB plate and incubating overnight at 37°C. The BW4005.1 clone was then inoculated into 10 ml LB and grown overnight at 37°C. This culture was used for genomic DNA extraction as described [29]. Whole genome re-sequencing of BW4005.1 was performed by 454 and single-end Solexa technologies [33]. Briefly, a total of 216,058 single-end reads with an average length of 404 bps, giving a 19.08X coverage of the genome, were generated using the GS FLX system (Roche, Welwyn Garden City, Hertfordshire, UK) and assembled to 78 contigs with 454 Newbler assembler (http://www.454.com/products/analysis-software/). A total of 8,862,831 single-end reads with an average length of 33 bps were generated using the Solexa Genome Analyzer (Illumina, Little Chesterford, Essex), giving a 63.9× coverage. All Solexa reads were mapped to the contigs generated from Newbler using MAQ [52]. The order of the contigs was determined based on BLASTn alignments with the genome sequence of BW2952 as the reference (Genbank accession number NC_012759). By analyzing these data, we found a few sequencing errors in the original BW2952 genome sequence (Additional file 1: Table S1). All 78 sequence gaps in BW4005 and 151 suspected SNP sites were filled or checked by sequencing PCR products with ABI 3730XL capillary sequencer (Applied Biosystems, Foster City, California, USA).

In our second genome re-sequencing attempt, a second colony derived independently from the same initial glycerol stock of BW4005, and called BW4005.C6 (see below, Table 4), was grown as described above. (BW4005.C6 was confirmed by our molecular analyses to harbor the heterozygous duplication). Its genomic DNA was extracted, checked and re-sequenced on the Illumina Genome Analyzer platform using single-end 36-bp reads (GATC-Biotech, Germany). Candidate mutations were identified in comparison to the ancestral BW2952 genome [29] using a recently developed computational pipeline [17]. Genome sequencing data have been deposited in the ENA Sequence Read Archive (ERP002251).

### RFLP-IS and characterization of IS adjacent sequences by inverse PCR

Restriction fragment length polymorphism (RFLP) using internal fragments of IS*3* and IS*5* as probes (RFLP-IS*3* and RFLP-IS*5*, respectively) was performed with genomic DNA of BW2952, BW4005 and derived clones as described previously [27]. Genomic sequences adjacent to relevant IS*3* elements were identified by inverse PCR experiments [27], using primers IS*3*ER and IS*3*EF (Table 2) that are complementary to the extremities of IS*3* and oriented toward its flanking regions. Briefly, after *Eco*RV digestion of genomic DNA from clone BW4005.2 and fractionation by agarose gel electrophoresis, the relevant DNA fragments were extracted from the gel, self-ligated and submitted to PCR using the primers IS*3*ER and IS*3*EF. The obtained PCR products were then sequenced to identify the IS*3* genomic neighborhood.

### Detection of the junction fragment of the duplication and analysis of the duplication stability

The new junction fragment generated by the duplication event was detected by PCR experiments using the two primer pairs FrontR/FrontF and IS*5*F1/IS*5*R1 (Table 2). The stability of the duplication was assessed by sub-culturing the evolved clone BW4005 in two different environments: non-selective LB medium and the chemostat evolution conditions. For non-selective conditions, the initial glycerol stock of BW4005 was plated onto LB solid media and incubated overnight at 37°C. Six colonies were sampled and grown for 12 hours in LB liquid medium at 37°C, the resulting clones being called BW4005.C1 to BW4005.C6. The BW4005.C6 clone, that was shown to retain the duplication after culture, was further inoculated by a 1:100 dilution into two independent McCartney bottles, each containing 5 ml LB, and incubated for 24 hours at 37°C with shaking at 200 rpm. These dilution/growth cycles were repeated for a total of three times. At each of the three steps, 100 μl of the appropriately diluted 24-hour cultures were plated onto LB solid medium and 32 well-separated colonies, 16 deriving from each of the two independent McCartney bottles, were sampled for DNA extraction and subsequent PCR analysis to score for the presence or absence of the duplication. For selective chemostat conditions, glucose-limited chemostats of BW4005.C6 and MC4100 were mixed after 16 hours of acclimation. Owing to the negative frequency-dependent fitness and low frequency of BW4005 in the initial chemostat evolution experiment [19], we mixed BW4005.C6 with MC4100 at a ratio of 1:99 and this co-culture was maintained for three days. Once every day, we sampled 32 colonies derived from BW4005.C6 after plating appropriate dilutions on LB plates and scoring for colonies resistant to kanamycin.

### Pulsed-field gel electrophoresis (PFGE)

PFGE was performed using a CHEF Mapper system (Bio-Rad Laboratories, Hercules, CA, USA) according to the procedure described by the US CDC PulseNet program (http://www.cdc.gov/pulsenet/pathogens/pfge.html). *Xba*I-digested genomic DNAs of BW2952, BW4005 and derived clones were separated at 6.0 V/cm for 19 hours at 14°C, with increasing pulse times of 6–36 s and an angle of 120°.

## Abbreviations

PFGE: Pulsed-field gel electrophoresis; RFLP: Restriction fragment length polymorphism; RFLP-IS3: Restriction fragment length polymorphism with IS*3* as probe; RFLP-IS5: Restriction fragment length polymorphism with IS*5* as probe; SNP: Single-nucleotide polymorphism.

## Competing interests

The authors declare that they have no competing interests.

## Authors’ contributions

RM carried out the physiological and fitness assays on all clones and constructed knockout strains. JG performed the RFLP-IS experiments. JG and JP constructed mutant strains. MS performed PFGE experiments. LW and LF were responsible for the genome sequencing and comparative genomics studies of BW4005.1. OT analyzed the genome sequence of BW4005.C6. DS and TF conceived the study, its design and coordination, and wrote the manuscript. All authors read and approved the final manuscript.

## Supplementary Material

Additional file 1: Table S1 lists the sequencing errors that were detected in the genome of the ancestor BW2952 that was previously sequenced [29]. Click here for file
