# Supplementary material for: A case of adaptation through a mutation in a tandem duplication during experimental evolution in Escherichia coli
Source: BMC Genomics. 2013 Jul 3;14:441. doi: 10.1186/1471-2164-14-441 (PMC3708739; doi:10.1186/1471-2164-14-441)
Supplement: Additional file 1: Table S1 — lists the sequencing errors that were detected in the genome of the ancestor BW2952 that was previously sequenced [29]. [file 1471-2164-14-441-S1.doc]

**Additional file**

**A case of adaptation through a mutation in a tandem duplication during experimental evolution in *Escherichia coli***

**By:** Ram P Maharjan, Joël Gaffé, Jessica Plucain, Lei Wang, Lu Feng, Olivier Tenaillon, Thomas Ferenci and Dominique Schneider

**Table S1 List of sequencing errors reported in the deposited genome sequence of the ancestral strain BW2952 [47]**

| Position^a^ | Nucleotide in BW2952 | Corrected sequence^b^ | Location | Flanking gene names^c^ |
| --- | --- | --- | --- | --- |
| 1178949 | G | C | Intergenic | IS*1*_*insA*(101) *rssB*(80) |
| 4139083 | T | No T | Intergenic | BWG_3698(54) BWG_3699(164) |
| 4168988 | C | No C | Intergenic | BWG_3737(291) BWG_3738(10) |
| 4409445 | C | T | Intergenic | *pyrL*(127) *yjgH*(154) |
| 4409446 | G | C | Intergenic | *pyrL*(126) *yjgH*(155) |
| 4409447 | T | G | Intergenic | *pyrL*(125) *yjgH*(156) |

^a^The positions are given according to the deposited genome sequence of the ancestor strain BW2952 [[47](#_ENREF_30)].

^b^The corrected sequences were obtained after Solexa re-sequencing of BW4005.C6 and confirmed by PCR/Sanger sequencing of the corresponding DNA regions of BW2952.

^c^In each case, the names of the two genes surrounding the mutation are given together with the distance, in bp, of the mutation from the start or the end of each of the corresponding genes.
